# Health Profile of Precarious Migrants Attending the Médecins Du Monde’s Health and Social Care Centres in France: a Cross-Sectional Study

**DOI:** 10.3389/ijph.2021.602394

**Published:** 2021-08-11

**Authors:** Emeraude Halley, Joris Giai, Marielle Chappuis, Anne Tomasino, Roland Henaine, Laurent Letrilliart

**Affiliations:** ^1^ Collège universitaire de médecine générale, Université Claude Bernard Lyon 1, Univ. Lyon, Lyon, France; ^2^ Service de Biostatistique-Bioinformatique, Hospices Civils de Lyon, Pierre-Bénite, France; ^3^ Médecins du Monde, Paris, France; ^4^ Unité d’enseignement Libre Médecine Humanitaire et SAMU Social, Université Claude Bernard Lyon 1, Lyon, France; ^5^ Service de chirurgie cardiaque C, Hôpital Cardiologique Louis Pradel, Bron, France; ^6^ Research on Healthcare Performance (RESHAPE), INSERM U1290, Université Claude Bernard Lyon 1, Lyon, France

**Keywords:** migrants, chronic condition, prevention, screening, primary care

## Abstract

**Objective:** The present study aimed to compare the precarious migrants’ health problems managed in Médecins du Monde’s health and social care centres (CASO) with those of patients attending general practice in France.

**Methods:** We compared the most frequent health problems managed in the 19 CASO in metropolitan France with those of a national sample of usual general practice consultations, after standardisation for age and sex.

**Results:** Precarious migrants had fewer health problems managed per consultation than other patients (mean: 1.31 vs. 2.16), and these corresponded less frequently to chronic conditions (21.3% vs. 46.8%). The overrepresented health problems among CASO consultations were mainly headache (1.11% vs. 0.45%), viral hepatitis (1.05% vs. 0.20%), type 1 diabetes (1.01% vs. 0.50%) and teeth/gum disease (1.01% vs. 0.23%). Their underrepresented health problems were mainly lipid disorder (0.39% vs. 8.20%), depressive disorder (1.36% vs. 5.28%) and hypothyroidism (0.50% vs. 3.08%). Prevention issues were nominal in precarious migrants (0.16%).

**Conclusion:** Both chronic somatic and mental conditions of precarious migrants are presumably underdiagnosed. Their screening should be improved in primary care.

## Introduction

The number of migrants coming to the European Union (EU) has increased in recent decades and reached 4.4 million in 2017 [1]. The migration flows are due to sociopolitical factors (armed conflicts or persecutions), economic factors (unemployment or poverty), and ecological factors (climate change) [2]. The main countries that received migrants in 2017 were Germany (917,100 newly arrived), the United Kingdom (644,200), Spain (532,100), France (368,900), and Italy (343,400) [1]. The migrant population was estimated to be 22.3 million in the EU in 2017 [1] and 7.9 million in France in 2015 [3]. The first-time asylum seekers in the EU in 2018 came mostly from Syria, Afghanistan, and Iraq [4], all countries with recent or ongoing conflicts.

Lack of awareness of their rights and the complexity of administrative procedures hinder the access of precarious migrants to usual primary care [5]. In France, fee exemption statuses are available to help those on a low-income, including precarious migrants, attend usual general practice. They can also receive free dedicated medical and social assistance in the hospital setting [6]. In addition, several humanitarian organizations, such as the NGO Médecins du Monde (MdM), provide primary care to this population. In MdM’s healthcare centers, people can consult physicians, nurses, and social workers freely and without appointment. More than nine out of 10 attenders to these centers are migrants without health insurance coverage at the first consultation [7].

Previous studies on the health of precarious migrants have often focused on one specific disease [2], or sometimes on their overall health based on their self-perceived health status [8, 9] or on morbidity data [10]. However, to the best of our knowledge, no published study has compared the precarious migrants’ health problems with those of patients attending usual general practice. The aim of the study was to use data from MdM’s health and social care centers and general practices in France to make this comparison.

## Methods

### Data Sources

The MdM database gathered data from 21 health and social care centers distributed across the whole French territory, referred to as “healthcare, advice, and referral centers” (*Centre d’accueil de soins et d’orientation*, CASO). For the present study, data were collected from the medico-social records of patients consulting from 1 January 2011 to 31 December 2012, including patient characteristics (age, sex, health insurance, housing status, and occupational status), health problem (s) managed during the consultation, and any drug prescriptions. The data for usual general practice were collected during a national cross-sectional study (ECOGEN) conducted in 128 French general practices between November 28, 2011 and April 30, 2012 [11]. These included patient data (age, sex, and health insurance), health problem(s) managed during the consultation, and any drug prescriptions.

### Data Management

We used the full ECOGEN database and extracted data recorded between December 2011 and April 2012 from the MdM database. In both databases, health problems had been originally coded according to the International Classification of Primary Care (ICPC-2) [12]. Chronic health problems were identified using a complementary classification derived from the ICPC-2 [13]. The MdM sample initially included 27,848 consultations. Data from Corsica (1,171 consultations) and Cayenne (113 consultations) CASO were excluded to obtain data from metropolitan France, geographically consistent with the ECOGEN sample. All consultations of French patients (*n* = 876) or with social or paramedical workers (*n* = 10,123), and data from follow-up consultations (*n* = 642), were also excluded from the MdM sample. The ECOGEN sample initially included 20,613 consultations, among which home visits (*n* = 1,269) and consultations only for social problems (*n* = 172) were excluded. Finally, 14,923 in CASOs and 19,172 usual general practice consultations were analyzed.

### Data Analysis

We first performed a direct standardization of CASO consultations on the usual general practice data for age and sex, to address the different distributions of these variables in the two samples. We then compared the most frequent health problems managed in CASO with those managed in general practice, using the total number of consultations as the denominator for each sample. We also compared the distribution of health problems managed in the two samples according to body systems (ICPC-2 chapters) [12].

Quantitative variables were described using mean and standard deviation and compared using Student’s t-test. Qualitative variables were described using count and frequencies and compared using the Chi-squared test. *p*-values below 5% were considered statistically significant, and all analyses were performed using R Software version 3.5.0 and the R package ‘survey’ version 4.0 [14].

### Ethical and Regulatory Aspects

The MdM database has been declared to the French data protection commission (*Commission nationale de l’informatique et des libertés*–CNIL, No. 731657). The ECOGEN study was approved by the regional review board (*Centre de protection des personnes–CPP Sud-Est IV*, No. L11-149) and declared to the CNIL (No. 1549782).

## Results

CASO patients were younger (mean age at consultation: 33.5 years *vs*. 44.7 years) and more often male (60.2% *vs*. 42.5% of consultations) than general practice patients. All consultations to general practices were made by patients who had health insurance coverage, and 94.9% of CASO consultations were made by patients who did not. Among CASO consultations, 55.3% were made by patients who came from Africa and 21.2% from the EU, 25.3% by patients who had stable housing (Table 1), and 39.8% by patients who had been living in France for less than 3 months.

**TABLE 1:** Social and demographic characteristics of patients consulting in Médecins du Monde’s centers and in general practice in metropolitan France in 2011–12.

	CASO centers N = 14,923	General practice N = 19,172	*p*-value
Age group, n (%)	—	—	<0.001
0–14 years	1,471 (10.0)	3,179 (16.6)	—
15–44 years	10,000 (67.7)	5,764 (30.1)	—
45–74 years	3,196 (21.6)	7,808 (40.7)	—
≥75 years	96 (0.7)	2,421 (12.6)	—
Missing data	160 (1.0)	0	—
Mean age (SD), yrs	33.5 (15,9)	44.7 (24,9)	<0.001
Sex, n (%)	—	—	<0.001
Female	5,932 (39.8)	11,028 (57.5)	—
Male	8,967 (60.2)	8,144 (42.5)	—
Missing data	24 (1.6)	0 (0)	—
Health insurance, n (%)	—	—	<0.001
Standard	0 (0)	18,281 (95.3)	—
Universal medical insurance (CMU)	371 (2.8)	837 (4.4)	—
State medical aid (AME)	300 (2.3)	54(0.3)	—
None	12,451 (94.9)	0 (0)	—
Missing data	1801 (12.0)	0 (0)	—
Native region, n (%)	—	—	—
North africa	4,104 (27.5)	n/a	—
Sub-saharan africa	4,149 (27.8)	n/a	—
European union	3,162 (21.2)	n/a	—
Europe non-EU	1,230 (8.2)	n/a	—
Near and middle east	435 (2.9)	n/a	—
Asia	1,049 (7.0)	n/a	—
Oceania and America	132 (0.8)	n/a	—
Missing data	414 (2.7)	—	—
Housing situation, n (%)	—	—	—
Homeless	5,715 (40.5)	n/a	—
Emergency accommodation	2,631 (18.6)	n/a	—
Organization or charity hosting	2,189 (15.5)	n/a	—
Stable housing	3,568 (25.3)	n/a	—
Missing data	820 (5.5)	—	—
Occupation, n (%)	—	—	—
Yes	2,194 (14.7)	7,488 (39.0)	—
No	9,736 (65.2)	11,684 (60.9)	—
Missing data	2,993 (20.0)	0 (0)	—

### Health Problems Managed

A lower mean number of health problems were managed per consultation during CASO consultations than general practice consultations (1.31 vs. 2.16, *p* < 0.001). A chronic condition was less often managed in CASO consultations than in general practice consultations (21.3% vs. 46.8%, *p* < 0.001). The following health problems were more often managed in CASO consultations than in general practice: cough (1.99% vs. 1.32%), low back symptom/complaint (1.50% vs. 1.07%), epigastric pain (1.20% vs. 0.89%), headache (1.11% vs. 0.45%), viral hepatitis (1.05% vs. 0.20%), type 1 diabetes (1.01% vs. 0.50%), teeth/gum disease (1.01% vs. 0.23%), refractive error (0.93% vs. 0.04%), and feelings of anxiety (0.90% vs. 0.63%; Table 2). All the 25 most frequent health problems managed in general practice consultations were more frequent than in CASO consultations, especially lipid disorder (8.20% vs. 0.39%), depressive disorder (5.28% vs. 1.36%), hypothyroidism (3.08% vs. 0.50%), sleep disturbance (2.98% vs. 0.75%), and osteoarthrosis (2.71% vs. 0.51%). Health maintenance/prevention issues were much frequent in general practice and virtually absent in CASO (23.24% vs 0.16%; Table 3).

**TABLE 2:** Top 25 health problems managed (ICPC-2 codes) in Médecins du Monde’s centers consultations (14,923) and in general practice consultations (19,172) in metropolitan France in 2011–12, according to the ranking in Médecins du Monde’s centers.

	CASO^a^ centers (standardized data)		General practice^b^		*p*-value
	%	(95%CI)	%	(95%CI)	
Hypertension, uncomplicated (K86)	7.65	(6.99–8.31)	14.77	(14.29–15.25)	<0.001
Upper respiratory infection, acute (R74)	6.48	(5.98–6.98)	9.97	(9.55–10.39)	<0.001
Diabetes, non-insulin dependent (T90)	3.66	(3.18–4.13)	5.04	(4.73–5.34)	<0.001
Acute bronchitis/bronchiolitis (R78)	2.26	(1.94–2.58)	3.21	(2.96–3.46)	<0.001
Cough (R05)	1.99	(1.70–2.28)	1.32	(1.16–1.48)	<0.001
Asthma (R96)	1.57	(1.30–1.85)	1.76	(1.57–1.94)	0.02
Low back symptom/complaint (L03)	1.50	(1.23–1.78)	1.07	(0.92–1.22)	<0.001
Depressive disorder (P76)	1.36	(1.12–1.59)	5.28	(4.96–5.59)	<0.001
Back syndrome with radiating pain (L86)	1.24	(0.98–1.50)	2.07	(1.86–2.27)	<0.001
Abdominal pain, epigastric (D02)	1.20	(0.97–1.44)	0.89	(0.76–1.03)	0.001
Back syndrome without radiating pain (L84)	1.20	(0.96–1.44)	2.36	(2.15–2.58)	<0.001
Tonsillitis, acute (R76)	1.18	(0.97–1.40)	1.68	(1.50–1.86)	<0.001
Laryngitidis/tracheitis, acute (R77)	1.15	(0.93–1.36)	1.35	(1.18–1.51)	0.01
Headache (N01)	1.11	(0.89–1.33)	0.45	(0.36–0.55)	<0.001
Viral hepatitis (D72)	1.05	(0.83–1.27)	0.20	(0.14–0.27)	<0.001
Diabetes, insulin dependent (T89)	1.01	(0.77–1.26)	0.50	(0.40–0.60)	<0.001
Teeth/gum disease (D82)	1.01	(0.82–1.20)	0.23	(0.17–0.30)	<0.001
Osteoarthrosis of knee (L90)	1.00	(0.74–1.25)	1.19	(1.04–1.34)	0.02
Teeth/gum symptom/complaint (D19)	0.99	(0.80–1.18)	0.31	(0.23–0.39)	<0.001
Constipation (D12)	0.97	(0.75–1.18)	1.97	(1.78–2.17)	<0.001
Refractive error (F91)	0.93	(0.71–1.16)	0.04	(0.01–0.07)	<0.001
Feeling anxious/nervous/tense (P01)	0.90	(0.70–1.11)	0.63	(0.52–0.74)	<0.001
Influenza (R80)	0.90	(0.71–1.09)	1.46	(1.29–1.63)	<0.001
Hypertension, complicated (K87)	0.90	(0.65–1.14)	1.45	(1.28–1.61)	<0.001
Neck syndrome (L83)	0.86	(0.65–1.07)	0.95	(0.81–1.09)	0.5

a The health problems managed were missing in 4,501 precarious migrants’ consultations.

b The health problems managed were missing in two general practice consultations.

**TABLE 3:** Top 25 health problems managed (ICPC-2 codes) in Médecins du Monde’s centers consultations (14,923) and in general practice (19,172) in metropolitan France in 2011–12, according to the ranking in general practice.

	CASO^a^ centers (standardized data)		General practice^b^		*p*-value
	%	(95%CI)	%	(95%CI)	
Health maintenance/prevention (A98)	0.16	(0.06–0.26)	23.24	(22.65–23.84)	<0.001
Hypertension, uncomplicated (K86)	7.65	(6.99–8.31)	14.77	(14.29–15.25)	<0.001
Upper respiratory infection, acute (R74)	6.48	(5.98–6.98)	9.97	(9.55–10.39)	<0.001
Lipid disorder (T93)	0.39	(0.23–0.55)	8.20	(7.83–8.58)	<0.001
No disease (A97)	0.26	(0.17–0.36)	6.12	(5.78–6.46)	<0.001
Depressive disorder (P76)	1.36	(1.12–1.59)	5.28	(4.96–5.59)	<0.001
Diabetes, non-insulin dependent (T90)	3.66	(3.18–4.13)	5.04	(4.73–5.34)	<0.001
Acute bronchitis/bronchiolitis (R78)	2.26	(1.94–2.58)	3.21	(2.96–3.46)	<0.001
Hypothyroidism (T86)	0.50	(0.32–0.68)	3.08	(2.84–3.32)	<0.001
Sleep disturbance (P06)	0.75	(0.58–0.92)	2.98	(2.74–3.22)	<0.001
Osteoarthrosis (L91)	0.51	(0.33–0.70)	2.71	(2.48–2.94)	<0.001
Back syndrome without radiating pain (L84)	1.20	(0.96–1.44)	2.36	(2.15–2.58)	<0.001
Bursitis/tendinitis/synovitis NOS (L87)	0.32	(0.19–0.45)	2.19	(1.98–2.39)	<0.001
Anxiety disorder/anxiety state (P74)	0.80	(0.62–0.97)	2.10	(1.89–2.30)	<0.001
Back syndrome with radiating pain (L86)	1.24	(0.98–1.50)	2.07	(1.86–2.27)	<0.001
Constipation (D12)	0.97	(0.75–1.18)	1.97	(1.78–2.17)	<0.001
Esophagus disease (D84)	0.47	(0.32–0.62)	1.90	(1.71–2.10)	<0.001
Vitamin/nutritional deficiency (T91)	0.04	(0.00–0.08)	1.77	(1.58–1.95)	<0.001
Asthma (R96)	1.57	(1.30–1.85)	1.76	(1.57–1.94)	0.02
Osteoporosis (L95)	0.08	(0.00–0.15)	1.73	(1.55–1.91)	<0.001
Tonsillitis, acute (R76)	1.18	(0.97–1.40)	1.68	(1.50–1.86)	0.001
Atrial fibrillation/flutter (K78)	0.13	(0.04–0.23)	1.61	(1.43–1.79)	<0.001
Sinusitis, acute/chronic (R75)	0.49	(0.34–0.63)	1.57	(1.39–1.75)	<0.001
Gastroenteritis presumed infection (D73)	0.17	(0.09–0.25)	1.57	(1.39–1.74)	<0.001
Gastrointestinal infection (D70)	0.22	(0.11–0.33)	1.53	(1.36–1.71)	<0.001

a The health problems managed were missing in 4,501 precarious migrants’ consultations.

b The health problems managed were missing in two general practice consultations.

### Body Systems Involved

**FIGURE 1:**
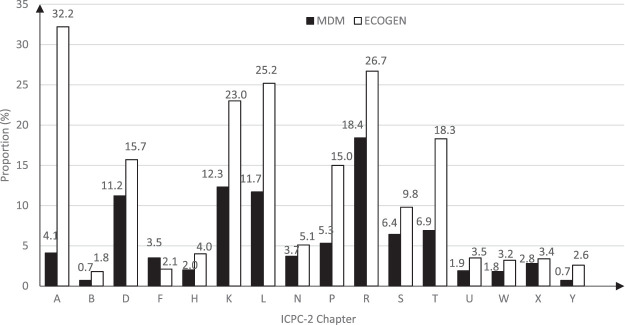
Distribution of health problems managed in precarious migrants’ and general practice consultations in metropolitan France in 2011–12 according to body systems (ICPC-2 chapters). The proportions of health problems managed in consultations in Médecins du Monde’s centers (black bars) and general practice consultations (white bars) are presented. A: General, B: Blood, D: Digestive, F: Eye, H: Ear, K: Cardiovascular, L: Musculoskeletal, N: Neurological, P: Psychological, R: Respiratory, S: Skin, T: Endocrine/Metabolic, U: Urological, W: Pregnancy/Family planning, X: Female genital, Y: Male genital. All comparisons between Médecins du Monde’s centers and general practice consultations were statistically significant. Since a consultation could contain several health problems, the total of the proportions exceed 100% in both samples.

Only eye problems (chapter F), dominated by refractive errors (47.2%), were more frequently managed in CASO consultations than in general practice (3.5% vs. 2.1%). Others were less frequent in CASO than in general practice, in particular psychological (5.3% vs. 15.0%), endocrine/metabolic (6.9% vs 18.3%), musculoskeletal (11.7% vs. 25.2%), and circulatory problems (12.3% vs. 23.0%). General and unspecified health problems (chapter A), including health maintenance/prevention issues, were less frequent in CASO consultations (4.1% vs. 32.2%; Figure 1).

### Drug Prescriptions

A drug was prescribed more often in CASO consultations than in general practice consultations (98.1% vs 80.4%, *p* < 0.001).

## Discussion

Consultations with precarious migrants mostly concerned young men living in difficult conditions related to poor housing and lack of health insurance coverage. These consultations included fewer health problems managed and a smaller proportion were chronic conditions. Overall, the most frequent health problems managed in usual general practice were less frequent in consultations with precarious migrants, especially prevention issues.

We observed that type 1 diabetes and viral hepatitis were more frequently managed in precarious migrants than in usual general practice patients, while the opposite was found for other chronic conditions. In the study sample, migrants mostly came from Africa or the EU, and those coming from Africa or Eastern Europe are prone to suffer from viral hepatitis B or C due to their endemic nature in these regions [15, 16]. Likewise, type 1 diabetes is expected to be frequent in precarious migrants, especially in those coming from the North-African countries [17]. Conversely, common health problems managed in usual French general practice, such as hypothyroidism, lipid disorders, and osteoarthrosis, were much less frequently managed in precarious migrants. Although the epidemiology of these chronic health problems is poorly documented in developing countries, they are presumably underdiagnosed in host countries because of their long silent course [18, 19]. Type 2 diabetes, which is common in the Middle East and North Africa [20], was also probably partly underdiagnosed. It is likely that CASO tend to focus more on acute health problems and to refer the management of chronic health problems to other healthcare professionals, which is supported by the relatively low number of medical consultations per patient (a mean 1.7 in 2017) [21].

In the present study, prevention issues were the most frequent health problems managed in usual general practice whereas they were nominal in CASO consultations; the quasi-systematic drug prescriptions in CASO is consistent with this finding. Precarious migrants can neglect preventive health issues because of competing priorities, such as housing and economic issues [22]. In addition, prevention can be underreported in CASO since preventive procedures are partly performed by dedicated nurses (health check) and partly referred to other healthcare facilities (usual general practices, screening for sexually transmitted infections and mother and child health) [21].

Anxiety was more often managed in CASO consultations than in usual general practice, while depressive disorders, and even more so, sleep disturbances were less frequently so. However psycho-traumas and depression are presumably highly prevalent among precarious migrants; the hazardous conditions of their migration and the discrimination or poverty they face in the host country can generate psychological problems [23, 24]. The findings herein therefore suggest that psychological problems are widely underdiagnosed in these patients. Such underdiagnosing can be favored by language and cultural barriers [25] and by the usually limited follow-up provided by the CASO [21].

With the exception of some chronic conditions, precarious migrants seemed in better apparent health than usual French general practice patients. We cannot exclude a possible healthy migrant effect, whereby those undertaking the often perilous migration routes are healthier than their compatriots [26]. Once settled in the host country, however, their health status tends to worsen due to socio-economic (unemployment) and environmental factors (poor housing and altered diet), although conditions of living and access to healthcare services may differ from country to country [27].

### Implications for Public Health

Underdiagnosis of health problems in precarious migrants could be reduced by improving screening procedures, as advised by several public health organizations [28—30]. The World Health Organization recommends ensuring healthcare access for newly arrived migrants and offering them health checks, including screening for communicable and non-communicable disease, without any further detail as to the types of screening [30]. The European Centre for Disease Prevention and Control has identified the following effective and cost-effective infections to be screened for: latent and active tuberculosis, HIV, hepatitis B and C, strongyloidiasis, and schistosomiasis [28]. These recommendations seem feasible and acceptable to migrants and healthcare professionals [31]. The French health authorities recommend providing newcomers with a comprehensive appointment including screening for communicable and non-communicable disease, as well as for history of physical and/or mental abuse and its health consequences [29]. These screening procedures should be performed while respecting migrants’ human rights, and the result should never be used as a reason for discrimination [30]. Disease detection should only be a first step and patients should be adequately managed thereafter. To ensure long-term continuity of care for migrants, a collaboration between first contact health centers, general practitioners, and other healthcare professionals is considered essential [28].

However, disease diagnosis and follow-up can be challenging in precarious migrants because of language differences, cultural background, and poor living conditions [32]. The language barrier can be alleviated with the support of telephone or face-to-face interpreting [33], but these services are rarely available in general practice and family members are often used for this purpose [34]. Communication strategies need to be adapted to the cultural and religious values of migrants, ideally by using dedicated documentation and with the help of cultural mediators [35, 36]. The management of psychiatric disorders is especially complex in migrants and requires referral to mental health professionals with cultural competence [32, 37].

### Strengths and Limitations

While confounding was controlled by the standardization of data, study limitations included risks of selection bias and more likely of information bias. The representativeness of the migrants attending CASO is supported by the participation of all French metropolitan CASO, and the representativeness of the GPs participating in the ECOGEN study has been validated previously for age, sex, practice location, and yearly number of consultations [20]. The anonymization of patient data in the ECOGEN database precluded the identification of those who consulted more than once during the study period. This constraint may have led to underestimating the number of health problems managed during each consultation with the same patient in general practice. However, the consequences are probably limited because of the five-month study period. Furthermore, the collection of data in the winter season probably generated an overrepresentation of respiratory infections, but similarly in both samples. The high proportion of missing health problems managed in CASO consultations may have introduced an information bias. These missing data were equally distributed in male and female patients but were slightly more frequent in young adults and less frequent in children (data not shown); yet, such bias was probably limited by the standardization of data.

### Conclusion

The lower frequency of chronic conditions and prevention issues managed in precarious migrants as compared to general practice patients suggests that chronic somatic and mental conditions are underdiagnosed. The access of precarious migrants to screening procedures should be improved based on guidelines, and adequately managed in primary care thereafter.

## Data Availability

The raw data supporting the conclusion of this article will be made available by the authors, without undue reservation.
